# British Association for the Advancement of Science

**Published:** 1839-10

**Authors:** 


### BRITISH ASSOCIATION FOR THE ADVANCEMENT OF SCIENCE.

The Association held its meeting this year at Birmingham, in the week beginning August 26. The proceedings in the medical section were, as usual, both important and interesting, and we regret that our limits will only allow us to give a mere catalogue of the papers redd.

#### Section E. Medical Science.

*President*—Dr. Yelloly. *Vice Presidents*—Dr. Johnstone, Dr. Roget, Dr. Macartney. *Secretaries*—Dr. G. O. Rees, Mr. F. Ryland. *Committee*—Drs. Blakiston, Booth, G. Bird; Mr. W. S. Cox; Drs. Evans, Foville, B. Fletcher, Hastings, T. Hodgkin; Mr. J. Hodgson; Drs. J. Johnstone, Pritchard; Mr. J. Russell; Drs. R. S. Sargent, Vose; Mr. R. Wood; Dr. Wale.

#### *List of Papers read.*

1. Abstracts of a remarkable case of Rupture of the Duodenum, and of some other interesting cases. By Sir David Dickson, M.D.
2. A notice of the methods that have been used for the removal of Capsular Cataract, where the opaque capsule remains after absorption of the Lens. By Mr. Middlemore.
3. Case in which the operation for Artificial Pupil was performed with success. By Mr. Middlemore.
4. On the means of repressing Hemorrhage from Arteries. By Professor Macartney.
5. On the Sounds produced in Respiration and on the Voice. By Dr. Blakiston.
6. Observations on Poisoning by the Vapours of Burning Charcoal. By Dr. Golding Bird.
7. On the Rules for finding with exactness the position of the principal arteries and nerves, from their relations to the external forms of the body. By Prof. Macartney.
8. On the Cause of the increase of Small-pox, and of the origin of Variola Vaccina. By Dr. Inglis.
9. On the New Vaccine Virus of 1838. By Mr. Estlin.
10. On Alkaline Indigestion. By R. D. Thomson, M.D.
11. On the Red Appearance of the Internal Coat of Arteries. By Mr. Hodgson.
12. On the Respiration of Deteriorated Atmospheres. By Mr. C. T. Coathupe.
13. Report of Ten Cases of Calculus treated by Lithotrity. By Mr. Costello.
14. On the Microscopic Structure of the Teeth. By Mr. Nasmyth.
15. On the Structure of the Epithelium. By Mr. Nasmyth.

The following grants were made to the section:

| | |
|---|---|
| Experiments on sounds of the heart . . . | £25 |
| Physiology of the lungs and bronchiæ . . . . | 25 |
| Construction of medico-acoustic instruments . . . | 25 |
| Enquiry into the connexion between the veins and absorbents | 25 |
| Experiments on acid poisons . . . . . | 25 |
| Total grants to Section E...... | £ 125 |

